# Peer Review of “Representing Physician Suicide Claims as Nanopublications: Proof-of-Concept Study Creating Claim Networks”

**DOI:** 10.2196/39859

**Published:** 2022-07-01

**Keywords:** physician suicide, suicide, suicide prevention, physician well-being, physician mental health, nanopublication, physician, doctor, mental health, semantic publishing, bibliometrics, claim network, information distortion, misinformation

*This is a peer-review report submitted for the paper “Representing Physician Suicide Claims as Nanopublications: Proof-of-Concept Study Creating Claim Networks.”*

## Round 1 Review

### General Comments

This paper [1] proposes a citation network of scientific publications about physician suicide. Such a citation network is a pioneering work for examining accurate claims of physician suicide. The network idea and entity schema design present unique values toward understanding the challenge.

### Specific Comments

#### Major Comments

1. Information completeness concerns: the authors claim that “A subset of articles from the literature search were identified that made an assertion (claim) about the annual rate of US physicians who die of suicide. Additional articles published between August 2019 and March 2020 have been identified and manually added to the article set used for this study.” However, such a data-searching procedure is not comprehensive and may lead to biased research. For example, the same source [2] of the Accreditation Council for Graduate Medical Education cited a paper back in 2003 with the same number, 300. If I did a google search or a professional database, I can find many more beyond the selected time periods. I would argue such an approach has a strong time bias and source bias. Do the authors conduct the investigation on a reliable database?

2. Nanopublication schema design: the schema is not well designed. For example, Figure 1 shows the number of fields is fixed and nonextensible. Therefore, that will lead each nanopublication to a limited citation size and a biased network. The authors may consider collaborations with scientists in a database or in computer science to redesign the toolkit. In addition, nanopublications can be revised or removed, and this design may lead to many false submissions. The authors may need to think about how to approach this because one contribution of this work is the toolkit.

#### Minor Comments

3. Some links are not accessible in the manuscript, such as [3].

4. The figures (eg, Figure 1) in the documents are quite blurry. The authors should consider using pictures with high resolutions.
